# Assessment of Computed Tomography Imaging for Isolated Type 1 Bicuspid Aortic Valve Repair: A Comparison between Internal and External Suture Annuloplasty Techniques

**DOI:** 10.31083/j.rcm2406174

**Published:** 2023-06-14

**Authors:** Qiming Ni, Liwen Fan, Wei Li, Shunan Ren, Xu Meng, Tianyang Yang

**Affiliations:** ^1^Department of Radiology, Shanghai Chest Hospital, Shanghai Jiao Tong University School of Medicine, 200030 Shanghai, China; ^2^Department of Thoracic Surgery, Renji Hospital, Shanghai Jiao Tong University School of Medicine, 200127 Shanghai, China; ^3^Department of Cardiac Surgery, Shanghai Chest Hospital, Shanghai Jiao Tong University School of Medicine, 200030 Shanghai, China; ^4^Department of Cardiac Surgery, Beijing Anzhen Hospital, Capital Medical University, 100029 Beijing, China; ^5^Department of Cardiac Surgery, Shanghai Forth People's Hospital, Tongji University School of Medicine, 200434 Shanghai, China

**Keywords:** bicuspid aortic valve, aortic valve repair, internal and external suture annuloplasty, computed tomography imaging

## Abstract

**Background:**

The ideal position of suture annuloplasty relative to the aortic annulus (internal or external) remains unclear. This study aimed to investigate the effectiveness of internal and external suture annuloplasty for isolated type 1 bicuspid aortic valve (BAV) repair. Electrocardiogram (ECG)-gated computed tomography (CT) was used to compare the two techniques and analyze their impact on the aortic annulus.

**Methods:**

We retrospectively analyzed 20 patients who underwent isolated type 1 BAV repair with either internal or external suture annuloplasty. Each group included 10 patients with comparable clinical features. Preoperative and postoperative ECG-gated CT scans were performed to assess the anatomical relationship between the ventricular-aortic junction (VAJ) and virtual basal ring (VBR), and to measure the height of annuloplasty from the VBR at predefined landmarks in both groups. Perioperative annular geometries, including annular area and perimeter, were measured to quantify the impact of annuloplasty on annular expansibility. The discrepancy between the postoperative annular dimension and size of the Hegar dilator were compared between groups to evaluate the effectiveness of annuloplasty.

**Results:**

In both groups, VAJ was higher than VBR at the right coronary (RC) ostium (7.7 ± 3.3 mm) and the raphe (7.9 ± 1.5 mm). The height from the VBR to the external suture annuloplasty shared a similar pattern at the RC ostium and raphe (5.3 ± 1.1 mm and 4.8 ± 1.0 mm, respectively). In contrast, the height differences were minimal for these landmarks in the internal group. Postoperative annular area expansibility decreased in the internal group compared to preoperative levels (4.9 ± 2.3% *vs.* 8.9 ± 5.5%, *p* = 0.038), while no significant change was found in the external group (7.6 ± 4.1% *vs.* 6.5 ± 2.8%, *p* = 0.473). The internal group showed less area discrepancy between the VBR and the Hegar dilator both at systole (10.1 ± 3.7% *vs.* 30.1 ± 16.6%, *p* = 0.004) and diastole (5.7 ± 4.9% *vs.* 20.9 ± 14.5%, *p* = 0.009) compared to the external group.

**Conclusions:**

Internal suture annuloplasty results in better positioning relative to the VBR than external suture annuloplasty due to the absence of VAJ interference. While this results in more precise annular reduction and less expansibility in the short term, a long-term follow-up evaluation is necessary to assess its effectiveness.

## 1. Introduction

The relationship between the effectiveness of annuloplasty and its position relative to the aortic annulus during isolated type 1 bicuspid aortic valve (BAV) repair remains unclear. This anatomical study aimed to compare internal and external suture annuloplasty and analyze their impact on aortic annulus type 1 BAV repair using computed tomography (CT).

Isolated BAV repair is a promising alternative to prosthetic valve replacement with reduced valve-related mortality and improved quality of life, which is especially meaningful for young patients with an active lifestyle and longer life expectancy [1, 2]. BAV is highly prevalent in younger patients (less than 50 years old) diagnosed with aortic regurgitation (AR), among whom Sievers’ type 1 with right and left cusp fusion (type 1 R/L) is the most common phenotype [3, 4]. Patients with isolated AR secondary to a dilated aortic annulus commonly present with BAV. One of the most important predictors of BAV repair failure is the lack of treatment for aortic annulus dilatations greater than 25–28 mm [5, 6, 7, 8].

Therefore, annuloplasty is of paramount importance in achieving annular stabilization and ensuring long-term durability of valve competency after BAV repair. Different techniques have been proposed to address annular dilatation, mainly classified as either external or internal annuloplasty based on how the annuloplasty devices are positioned towards the level of the virtual basal ring (VBR, the plane passing through the nadir of the aortic cusps) [6, 7, 8, 9, 10, 11, 12, 13, 14, 15, 16, 17]. External annuloplasty requires deep surgical dissection of the aortic root, where the ventricular-annular junction (VAJ) is anatomically higher than the VBR, to reach the nadirs of the aortic cusps, and such maneuvers are more challenging in isolated BAV repair, which requires extensive root preparation to secure the coronary arteries.

Both external and internal annuloplasty has been reported [7, 14, 18, 19, 20] with a technique utilizing an expanded polytetrafluoroethylene suture. An internal suture was placed inside the left ventricular outflow tract (LVOT) at the level of the VBR without root dissection. Although proper positioning of annuloplasty sutures at the desired level is crucial to achieve appropriate annular stabilization, very few imaging studies have assessed the exact position of different annuloplasty techniques relative to the VBR and the subsequent impact on annular morphology and dynamics, especially in BAV repair.

Our previous study showed that electrocardiogram (ECG)-gated CT were able to precisely measure the normal tricuspid aortic valve [21]. ECG-gated CT may similarly provide valuable information for isolated BAV repair by facilitating quantitative assessment of different annuloplasty techniques, thereby leading to a more standardized and reproducible approach.

The aim of this anatomical study was to compare external and internal suture annuloplasty in isolated type 1 R/L BAV repair and analyze their morphological features using CT reconstruction.

## 2. Materials and Methods

### 2.1 Patients

This study was approved by the Ethics Committee of Shanghai Chest Hospital (ethics number: IS23011), and the requirement for informed consent was waived because of the retrospective nature of the study.

From October 2021 to September 2022, 41 patients with BAV underwent aortic valve repair for AR with or without ascending aortic aneurysms at the Shanghai Chest Hospital. Among them, 20 patients with type 1 R/L who underwent isolated valve repair were retrospectively analyzed and classified into two groups: patients who underwent external suture annuloplasty (10 patients before April 2022) and patients who underwent internal suture annuloplasty (10 patients after April 2022). The remaining 21 patients were excluded based on the following criteria: patients without a complete diagnostic workup with adequate quality pre- and post- operative ECG-gated CT scans; concurrent aortic stenosis with more than moderate severity; patients with aortic root dilatation with a cut-off diameter of 45 mm or type A dissection involving the aortic root, thereby requiring additional root reimplantation; and BAV of subtypes other than type 1 R/L. The baseline characteristics of patients with preoperative echocardiographic data are presented in Table 1.

**Table 1.** **Clinical and perioperative echocardiographic features of patients underwent bicuspid aortic valve repair with internal or external annuloplasty**.

Variables			Internal group (n = 10, %)	External group (n = 10, %)	*p *value
Age (years, mean ± SD)			34.2 ± 7.3	30.0 ± 7.8	0.229
Gender					1.0
	Male		9 (90)	9 (90.0)	
	Female		1 (10)	1 (10.0)	
Height (cm, mean ± SD)			172.2 ± 7.5	173.6 ± 9.6	0.721
Weight (kg, mean ± SD)			76.5 ± 10.3	73.4 ± 16.0	0.613
Body surface area ($m^{2}$, mean ± SD)			1.99 ± 0.15	1.96 ± 0.25	0.748
Preoperative echocardiography					
	LVEF (%, mean ± SD)		63.3 ± 4.9	63.4 ± 3.8	0.960
	LVEDD (mm, mean ± SD)		64.2 ± 9.2	64.5 ± 8.8	0.941
	LVESD (mm, mean ± SD)		42.1 ± 8.0	40.3 ± 6.8	0.595
	LVEDV (mL, mean ± SD)		212.2 ± 68.7	221.2 ± 62.5	0.763
	LVESV (mL, mean ± SD)		80.1 ± 37.1	81.7 ± 28.0	0.915
	Root (mm, mean ± SD)		37.4 ± 4.0	39.6 ± 3.3	0.194
	STJ (mm, mean ± SD)		33.0 ± 5.8	32.2 ± 3.0	0.704
	Ascending aorta (mm, mean ± SD)		38.0 ± 8.6	36.7 ± 6.7	0.711
Aortic regurgitation					1.0
	Moderate		1 (10.0)	0 (0)	
	Severe		9 (90.0)	10 (100%)	
Pressure gradient (mmHg, mean ± SD)			12.4 ± 7.8	14.6 ± 8.9	0.564
CO angle (degree, mean ± SD)			144.2 ± 13.2	142.3 ± 12.9	0.748
Postoperative echocardiography					
	Aortic regurgitation				0.889
		Non	4 (40.0)	3 (30.0)	
		Trivial	5 (50.0)	6 (60.0)	
		Mild	1 (10.0)	1 (10.0)	
Pressure gradient (mmHg, mean ± SD)			15.6 ± 5.4	15.3 ± 4.5	0.894
CO angle (degree, mean ± SD)			159.5 ± 10.0	154.4 ± 13.9	0.358
Ascending aortic replacement					1.0
	Yes		2 (20.0)	3 (30.0)	
	No		8 (80.0)	7 (70.0)	
STJ remodeling					0.370
	Yes		4 (40.0)	7 (70.0)	
	No		6 (60.0)	3 (30.0)	
Hegar diameter (mm, mean ± SD)			22.1 ± 1.4	24.0 ± 1.4	0.008

SD, standard deviation; LVEF, left ventricular ejection fraction; LVEDD, left ventricular end-diastolic diameter; LVESD, left ventricular end-systolic diameter; LVEDV, left ventricular end-diastolic volume; LVESV, left ventricular end-systolic volume; STJ, sinotubular junction; CO, commissure orientation.

### 2.2 Surgical Procedure

The choice between the two annuloplasty techniques has evolved with time and surgical access. Before April 2022, full median sternotomy was routinely performed for surgical access in the external suture annuloplasty group. From then on, we turned to partial upper sternotomy for the group with internal suture annuloplasty, with less impact on chest wall integrity and a better cosmetic outcome, which is meaningful for young patients. However, it is more difficult to perform deep root dissection and hemostasis after recovery of heart beating for external annuloplasty. Therefore, we adopted the less commonly used internal suture annuloplasty approach to avoid these shortcomings.

After cardiopulmonary bypass was initiated, the aorta was cross-clamped, the heart was arrested, and the following steps were performed in the group with external suture annuloplasty. The aorta was transected 5 mm above the sinotubular junction. Commissural resuspension sutures are used to expose the aortic valve. Aortic valve leaflets were inspected for tissue quality and quantity by measuring the geometric height of the non-fused cusp and half of the fused R/L leaflet. The decision for aortic valve repair was based on a comprehensive evaluation of the degree of raphe fusion, commissural orientation, and leaflet mobility. The right coronary (RC) and left coronary (LC) origins were isolated but not detached from the sinus through blunt dissection with a right-angle clamp and were secured by a stay suture passing beneath the ostia. Dissection was performed externally along the aortic root between the right and left coronary origins. Due to variations in the level of the VAJ relative to the VBR and the frequent presence of “sinking sinus” in this area [22], we did not aim to reach a deep subvalvular plane to avoid extensive myocardial dissection, but only to create a proper space for passing the needle of suture annuloplasty externally, as suggested in the literature [6]. The root dissection was continued along the left and non-coronary (NC) sinuses posterior to the level of the VBR, which was easily accomplished with routine dissection, as the VAJ is either almost at the same level as the VBR or absent where the curtain is located. Annulus dilatation, defined as a diameter >25 mm measured in a preoperative imaging study and confirmed intraoperatively with a Hegar dilator, was treated by external circular suture annuloplasty using a CV-0 polytetrafluoroethylene single-needle suture (W. L. Gore & Associates, Newark, DE, USA) according to Schaf̈ers’ technique [6, 14]. We aimed to place the suture deep while avoiding injury to the surrounding structures.

In the internal suture annuloplasty group, aortic root dissection was not required between the right and left coronary origins. Similar to the technique described by Holst *et al*. [23], following the VBR plane under direct vision, the suture was started from inside the left ventricular outflow tract and passed outside the area of the NC sinus. Care was taken to elevate the internal suture line along the membranous region to avoid injury to the conduction system.

At all times, the sutures were tightened across the aortic annulus using a Hegar dilator. The sizing strategy with the Hegar dilator was more aggressive, with a smaller diameter in the internal group to increase coaptation length (see Table 1 for Hegar diameter). Thus, we aimed to delay the recurrence of AR in the event of postoperative annular redilatation. This is particularly important considering there is limited evidence of the effectiveness of annular stabilization using the internal suture technique.

Leaflet repair procedures were performed to achieve valve competency in both groups. The effective height of the cusp was assessed with a caliper, and any cusp prolapse was corrected by free margin plication with 5-0 polypropylene suture to obtain an equivalent free margin of both leaflets with an effective height of 9–10 mm.

### 2.3 CT Imaging Protocol and Measurements

Contrast-enhanced ECG-gated CT of the aortic root was performed in both groups 2–3 days before and 5–7 days after surgery. The CT protocol and image reconstruction methods were introduced in our previous study [21]. All CT data were systematically analyzed using Osirix software version 9.5.1 (Pixmeo, Geneva, Switzerland). Multiplane reconstruction was performed to visualize the planes of interest (VBR and VAJ) for BAV repair.

### 2.4 Definition and Measurement of VBR

**Fig. 1.**
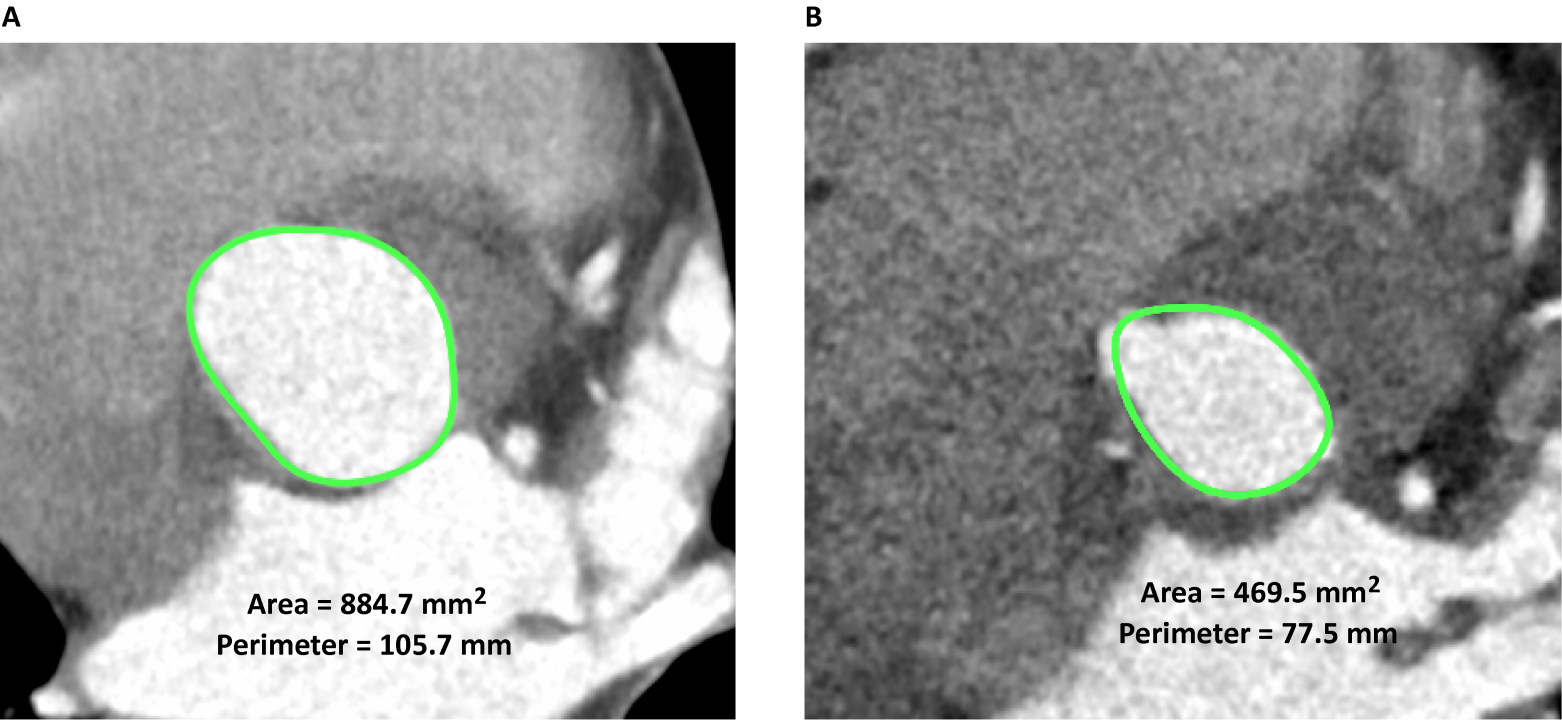
**Definition and measurement of VBR by reconstructed ECG-gated computed tomography**. The axial image passing through the nadir in the NC sinus and in the midportion of the respective half of the fused anterior leaflet (approximated nadir of each fused leaflet) was identified as the VBR in type 1 R/L BAV. The pictures show the annular area and perimeter before BAV repair in systole (A) and diastole (B) at 20% and 80% of the R-R interval (green circle). VBR, virtual basal ring; ECG, electrocardiogram; BAV, bicuspid aortic valve; NC, non-coronary.

Through dedicated multiplanar reconstruction with the application of a double oblique view, an axial plane perpendicular to the long axis of the aortic root was obtained. The axial image passing through the nadir in the NC sinus and the midportion of the respective half of the fused anterior leaflet (approximate nadir of each fused leaflet) was identified as the VBR in type 1 R/L BAV. In both groups, preoperative and postoperative perimeters and areas were measured during systole and diastole at 20% and 80% of the R-R interval, respectively [24] (Fig. 1).

### 2.5 Definition of VAJ and its Topographic Relationship with VBR

**Fig. 2.**
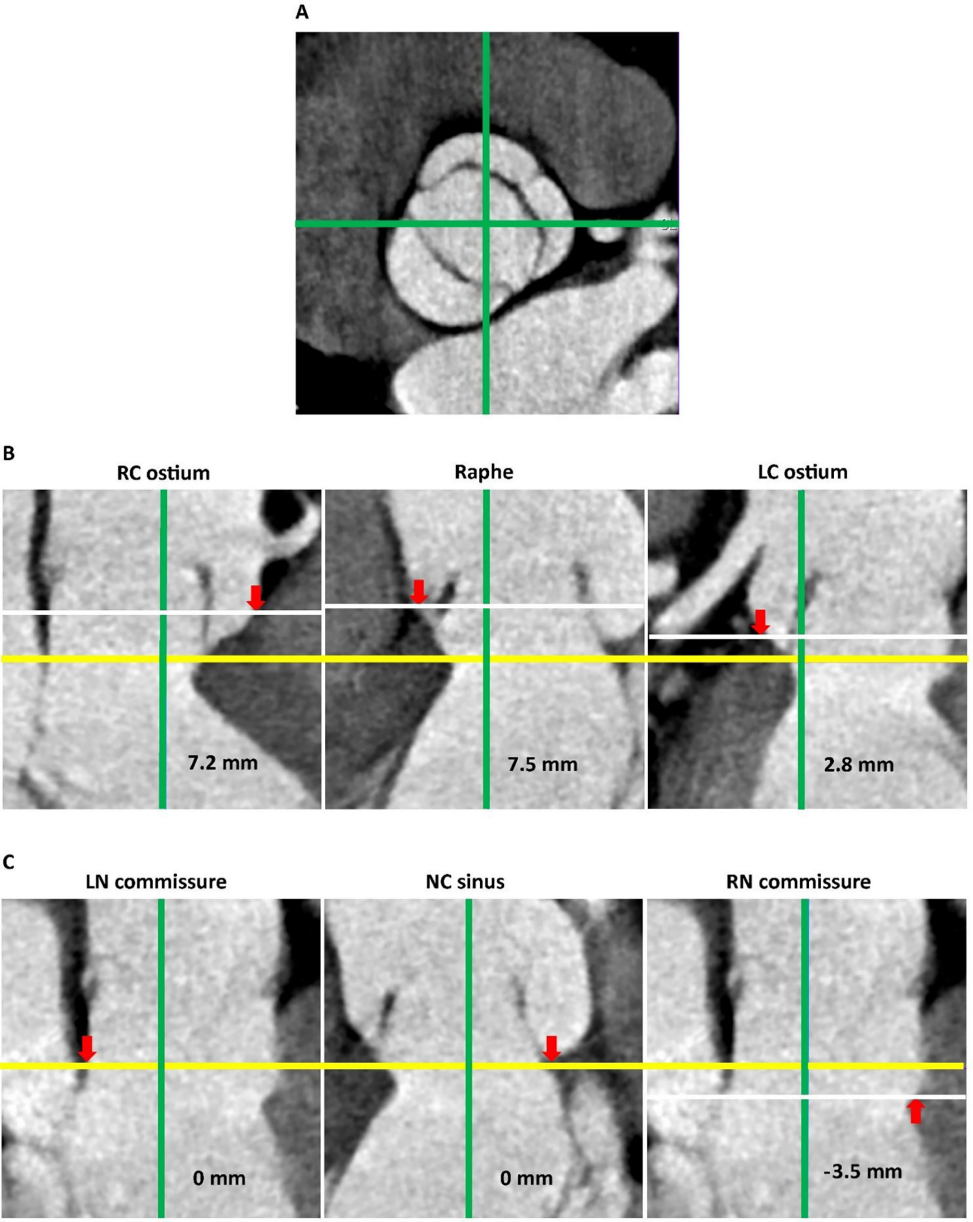
**Identification of VAJ and measurement of heights from VBR at predefined anatomical landmarks**. (A) The short axis view of the aortic root. Anatomical landmarks are identified on short axis view of the aortic root by rotation of 2 orthogonally-crossed plane perpendicular to the VBR. Green line: orthogonally-crossed planes in the long axis view of the aortic root. (B) Height of VAJ from VBR in the long axis views of the aortic root along the anterior aortic annulus. (C) Height of VAJ from VBR in the long axis views of the aortic root along the posterior aortic annulus, the white line did not appear in the first and second pictures as it coincides with the yellow line. Yellow line: level of the VBR. White line: level of the anatomical landmarks. Red arrows: position of anatomical landmarks. VAJ, ventricular-aortic junction; VBR, virtual basal ring; RC, right coronary; LC, left coronary; LN, left-non; NC, non-coronary; RN, right-non.

According to the anatomic study, the VAJ was more of a circular structure consisting of different tissue components (interventricular septum, aortomitral curtain, and connective tissue) rather than a planar circle strictly above the VBR, as initially thought. Owing to its three-dimensional curvilinear configuration, the height of the VAJ relative to the VBR varies along the root circumference. We measured these heights in 6 long axis views of aortic root perpendicular to the VBR plane at 20% of the R-R interval preoperatively, with each view corresponding to a specific predefined landmark of the aortic root circumference. These six landmarks were set at the nadir in the NC sinus, RC and LC ostium, right/non (RN) and left/non (LN) commissure, and non-functional commissure adjacent to the raphe of the fused cusp (raphe) (Fig. 2).

### 2.6 Identification of Suture Annuloplasty and its Topographic Relationship with VBR

**Fig. 3.**
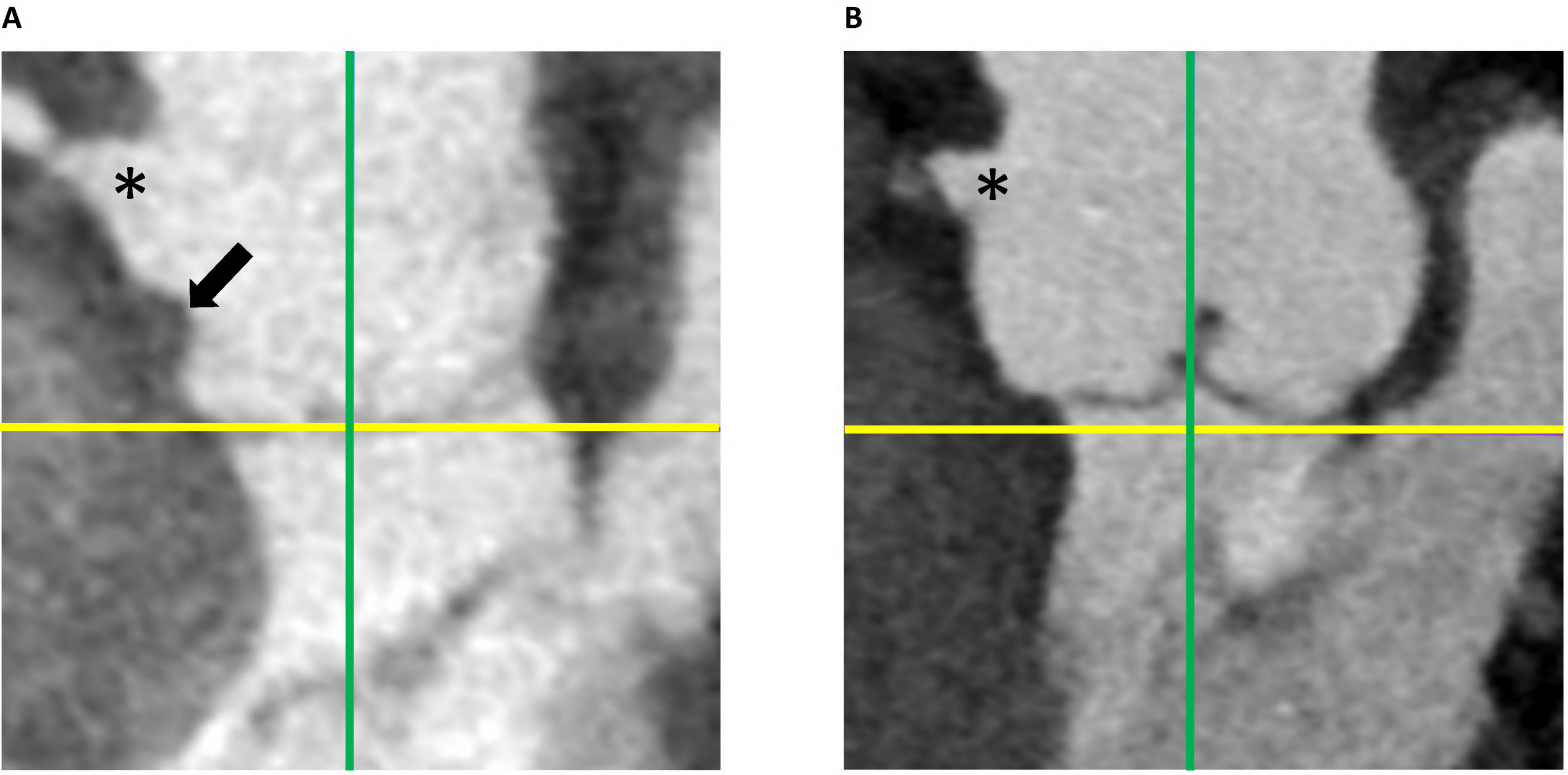
**Postoperative long axis views of the aortic root after BAV repair**. (A) Waist sign caused by the narrowing effect of the external suture annuloplasty. (B) No waist sign was seen by BAV repair with internal suture annuloplasty. Yellow line: level of the virtual basal ring. Green line: orthogonally-crossed planes in the long axis view of the aortic root. The asterisk: right coronary ostium. Black arrow: waist sign. BAV, bicuspid aortic valve.

The CV-0 suture was not radiopaque; therefore, the annuloplasty positioning was assessed based on the presence of a characteristic narrowing effect induced by tightening the suture within or outside the aortic root on postoperative CT images. The level of suture positioning, either higher or lower than the VBR plane, produces a notch over the aortic root or LVOT on long-axis views of the aortic root perpendicular to the VBR plane. This narrowing effect is a feature of root distortion, referred to as the waist sign (Fig. 3A). The distance between the waist sign and VBR plane was measured in the same manner as the height of the VAJ using the aforementioned landmarks (Fig. 2). The distance between the annuloplasty suture and the VBR was considered as 0 if no waist signs were identified (Fig. 3B).

### 2.7 Statistical Analysis

Measurement data were assessed to compare different patient groups or subgroups using the chi-square test and Fisher’s exact probability test for categorical variables and the two-tailed Student’s *t*-test for continuous variables. Continuous variables were summarized as mean and standard deviation. Categorical variables were expressed as numbers and percentages. A *p*-value of <0.05 was considered statistically significant. All analyses were conducted using SPSS software (version 22.0; IBM Corporation, Chicago, IL, USA).

## 3. Results

### 3.1 Preoperative Relationship between VAJ and VBR Plane in Type 1 R/L BAV

The height of VAJ related to the VBR circumference was uneven with the highest at the raphe and RC ostium (7.9 ± 1.5 mm, 7.7 ± 3.3 mm, respectively), and VAJ is at the same level as the VBR at the NC sinus and LN commissure. The height of the RC and LC ostia from the VBR and the distance between them, both of which are related to the complexity of anterior annulus dissection, were also assessed. There were no significant differences between the internal and external groups in any of these parameters (Table 2).

**Table 2.** **Preoperative computed tomographic features of patients underwent bicuspid aortic valve repair with internal or external annuloplasty**.

Variables (mm, mean ± SD)		In total (n = 20)	Internal group (n = 10)	External group (n = 10)	*p *value
Distance between LC and RC ostium		25.5 ± 7.8	22.3 ± 8.3	28.8 ± 5.9	0.057
Mean coronary ostium height from VBR plane					
	LC	13.6 ± 2.6	12.9 ± 2.7	14.3 ± 2.4	0.224
	RC	17.0 ± 3.1	16.7 ± 3.5	17.4 ± 2.7	0.610
Mean VAJ height from VBR plane at different land marks					
	LC ostium	2.4 ± 0.9	2.3 ± 0.8	2.5 ± 1.0	0.702
	RC ostium	7.7 ± 3.3	7.2 ± 2.6	8.2 ± 4.0	0.524
	RN commissure	–0.39 ± 1.7	–0.56 ± 1.6	–0.21 ± 1.9	0.658
	LN commissure	0	0	0	/
	Raphe	7.9 ± 1.5	7.9 ± 1.2	7.8 ± 1.9	0.889
	NC sinus	0	0	0	/

SD, standard deviation; LC, left coronary; RC, right coronary; VBR, virtual basal ring; VAJ, ventricular-aortic junction; RN, right-non; LN, left-non; NC, non-coronary.

### 3.2 Postoperative Relationship between Suture Annuloplasty and VBR Plane

Results from the external group showed that the suture annuloplasty was farthest from the VBR along the anterior annulus at the RC ostium (5.3 ± 1.1 mm), followed by the raphe (4.8 ± 1.0 mm). Conversely, the suture was closest to the VBR at the LC ostium (1.9 ± 1.7 mm), LN commissure (2.2 ± 1.8 mm), and NC sinus (2.3 ± 1.5 mm), where VAJ was also closer to the VBR plane. The internal group showed minimal identification of waist sign, except at the RN commissure, where sutures deviated the most from the VBR, as seen in the external group (4.3 ± 1.4 mm *vs.* 5.8 ± 1.5 mm, *p* = 0.026). This deviation was intended to prevent injury to the conduction system in both groups. However, the internal group had significantly shorter distances between the suture annuloplasty and the VBR than the external group at all landmarks (Table 3).

**Table 3.** **Postoperative computed tomographic features of patients underwent bicuspid aortic valve repair with internal or external annuloplasty**.

Variables (mm, mean ± SD)		Internal group (n = 10)	External group (n = 10)	*p *value
Mean suture annuloplasty height from VBR plane at different land marks				
	LC ostium	0.10 ± 0.3	1.9 ± 1.7	0.009
	RC ostium	0.27 ± 0.6	5.3 ± 1.1	<0.001
	RN commissure	4.3 ± 1.4	5.8 ± 1.5	0.026
	LN commissure	0	2.2 ± 1.8	0.004
	Raphe	–0.3 ± 0.7	4.8 ± 1.0	<0.001
	NC sinus	0.45 ± 0.6	2.3 ± 1.5	0.002

SD, standard deviation; VBR, virtual basal ring; LC, left coronary; RC, right coronary; RN, right-non; LN, left-non; NC, non-coronary.

### 3.3 The Effect of Annuloplasty on the Geometry of VBR

The preoperative geometrical parameters of VBR were comparable between the two groups during the cardiac cycle. Both groups showed a significant reduction in the systolic and diastolic annular dimensions after annuloplasty (Table 4). To account for the different annular reduction strategies between the groups (smaller Hegar dilator diameter used in the internal group compared to the external group, 22.1 ± 1.4 mm *vs.* 24.0 ± 1.4 mm, *p* = 0.008), postoperative VBR was not directly analyzed. Instead, the effect of annuloplasty was assessed by comparing annular expansibility (variation in area and perimeter between systolic and diastolic sequences) and the dimensional discrepancy between postoperative VBR and Hegar dilator between the two groups. Postoperative annular area expansibility decreased in the internal group compared to preoperative levels (4.9 ± 2.3% *vs.* 8.9 ± 5.5%, *p* = 0.038) with a trend of decreased perimeter expansibility (2.3 ± 2.4% *vs.* 3.6 ± 3.9%, *p* = 0.259), while no significant change in expansibility was found in the external group. The internal group showed less discrepancy between the VBR and Hegar dilator, both in area and perimeter, at systole and diastole compared with the external catheter group (Table 5).

**Table 4.** **Perioperative computed tomographic features of VBR of patients underwent bicuspid aortic valve repair with internal or external annuloplasty**.

Variables		Internal group			External group			Preoperative *p *value between groups	Postoperative *p *value between groups
		Preoperative	Postoperative	*p *value	Preoperative	Postoperative	*p *value		
Systole									
	Area (${\mathrm{mm}}^{2}$, mean ± SD)	829.4 ±155.3	424.2 ± 57.7	<0.001	870.7 ± 219.7	588.8 ± 95.9	<0.001	0.634	/
	Perimeter (mm, mean ± SD)	102.5 ± 10.5	74.1 ± 5.3	<0.001	105.5 ± 12.5	87.7 ± 7.1	<0.001	0.570	/
Diastole									
	Area (${\mathrm{mm}}^{2}$, mean ± SD)	760.0 ± 128.5	404.7 ± 56.2	<0.001	815.3 ± 194.1	547.3 ± 85.5	<0.001	0.463	/
	Perimeter (mm, mean ± SD)	98.9 ± 8.4	72.4 ± 4.9	<0.001	101.7 ± 11.8	84.9 ± 6.8	<0.001	0.545	/
Expansibility									
	Mean area variation (%)	8.9 ± 5.5	4.9 ± 2.3	0.038	6.5 ± 2.8	7.6 ± 4.1	0.473	0.225	0.078
	Mean perimeter variation (%)	3.6 ± 3.9	2.3 ± 2.4	0.259	3.7 ± 1.6	3.3 ± 1.5	0.474	0.935	0.272

VBR, virtual basal ring; SD, standard deviation.

**Table 5.** **Postoperative difference between VBR dimension and Hegar size during the cardiac cycle: comparison in each group and between groups**.

Variables		Internal group					External group					Systole *p* value between groups	Diastole *p* value between groups
		Systole	Diastole	${\mathrm{Hegar}}^{1}$	*p* value		Systole	Diastole	${\mathrm{Hegar}}^{2}$	*p* value			
					Systole *vs.* ${\mathrm{Hegar}}^{1}$	Diastole *vs.* ${\mathrm{Hegar}}^{1}$				Systole *vs.* ${\mathrm{Hegar}}^{2}$	Diastole *vs.* ${\mathrm{Hegar}}^{2}$		
${\mathrm{Area}}^{3}$ (${\mathrm{mm}}^{2}$, mean ± SD)		424.2 ± 57.7	404.7 ± 56.2	384.9 ± 48.2	<0.001	0.010	588.8 ± 95.9	547.3 ± 85.5	453.6 ± 52.9	<0.001	0.002	**/**	**/**
${\mathrm{Perimeter}}^{4}$ (mm, mean ± SD)		74.1 ± 5.3	72.4 ± 4.9	69.4 ± 4.6	<0.001	<0.001	87.7 ± 7.1	84.9 ± 6.8	75.4 ± 4.4	<0.001	<0.001	**/**	**/**
Discrepancy between VBR and Hegar													
	Mean Area ${\mathrm{difference}}^{3}$ (% ± SD)	10.1 ± 3.7	5.7 ± 4.9	/	/	/	30.1 ± 16.6	20.9 ± 14.5	/	/	/	0.004	0.009
	Mean Perimeter ${\mathrm{difference}}^{4}$ (% ± SD)	6.8 ± 1.6	4.8 ± 2.4	/	/	/	16.5 ± 7.5	12.7 ± 7.3	/	/	/	0.003	0.008

VBR, virtual basal ring; SD, standard deviation; ^1^, used for the internal procedure; ^2^, used for the external procedure; ^3^, The area of Hegar was calculated from the diameter using the circle formula; ^4^, The perimeter of Hegar was calculated from the diameter using the circle formula.

## 4. Discussion

The crucial role of annuloplasty in repairing BAV to treat AR has been extensively studied and reported [1, 2, 14, 18, 22]. In isolated BAV repairs, annular dilatation has been identified as a standalone risk factor for the recurrence of regurgitation [25, 26]. When annular dilatation is present before surgery, adding annuloplasty to isolated BAV repair aims to reduce or stabilize the dimensions of the annulus. This in turn enhances the long-term durability of valve competency.

Annular dilatation is almost omnipresent in type 1 R/L BAV with AR and is characterized by the anatomical interaction between the VBR and surrounding VAJ. Due to the transition of the ventricular myocardium to the aortic wall, the VAJ has variable height and thickness along the circumference of the VBR, formed by the plane passing the nadirs of each cusp, which leads to a difference in location between the VBR and VAJ [27]. In view of the complex annular anatomy and its close interplay with important neighboring structures (coronary ostia and conduction system), different annuloplasty techniques have been proposed over time by different groups, all of which aim for the VBR, rather than the VAJ, as the target of annular reduction/stabilization. The external ring annuloplasty suggested by Lansac *et al*. [2, 7, 8] requires the creation of tunnels under both the coronary ostia and root dissection, similar to the reimplantation technique, along the anterior annulus to seat the ring at an optimum level. Improved repair stability has been reported with the use of this technique in isolated BAV repair [2]. However, a study using CT imaging revealed that the Lansac ring was still partially above the VBR, especially at the level of the commissure between the left and right coronary sinuses in the tricuspid aortic valve non-functional commissure in type 1 R/L BAV and at the level of the right coronary sinus [28]. According to de Kerchove *et al*. [27], if the lowest point of the right coronary sinus is not reached during dissection of the anterior annulus, especially in type 1 R/L BAV, where sinking sinuses are more prevalent, the annuloplasty ring or proximal reimplantation graft may have a tilted basal attachment. This can result in insufficient annular support and potentially impair the long-term durability of the repair. They suggested that deep anterior dissection averts this problem; however, breaching of the right ventricular cavity is inevitable in some cases, and a higher rate of pacemaker use was observed. According to an imaging study conducted by Irace *et al*. [29], despite aggressive deep dissection during the reimplantation procedure, the base of the graft, which serves as the supporting annuloplasty site, remains seated on the VAJ at varying thicknesses and heights along the VBR circumference. This finding was consistent with a previous study [27]. Schneider *et al*. [6, 14] have described both external and internal suture annuloplasty techniques that do not require deep root dissection and have been shown to have a very low rate of surgical complications. However, there is no imaging evidence showing the actual position of these sutures in relation to VBR.

Inspired by a previous study [27] in which the topographic relationship between the VAJ and VBR was quantified on cadaver root specimens in an *in vitro* setting, we performed a similar assessment of type 1 R/L BAV with AR using reconstructed multislice (MS)-CT images. To the best of our knowledge, our study is the first to use ECG-gated MS-CT imaging to investigate BAV of an identical phenotype and perform measurements under *in vivo* human conditions. The unique nature of our study makes it highly valuable as it provides insight into a better understanding of annuloplasty in BAV repair. Analysis of the preoperative BAV images revealed that the VAJ was above the VBR mainly around the anterior annulus, with significant height differences at points corresponding to the RC ostium, raphe, and LC ostium (Table 1). Interestingly, the same pattern was observed for height differences between VBR and external suture annuloplasty in the postoperative images of the external group (Table 2). On the contrary, such phenomenon was not identified in the internal group, which reflected a better overlapping with VBR using internal suture annuloplasty. The different effect of the two techniques could be explained by the following reasons: (1) during internal suture annuloplasty, the nadir of the cusps and the base of the inter-commissural triangle can be identified under direct vision; therefore, the suture can pass at the exact level of VBR formed by these anatomical landmarks; (2) performing external annuloplasty within the VAJ around the anterior annulus without deep root dissection can be challenging. The myocardium above the VBR and the nondetached coronary artery make it difficult to maintain a consistent plane during each suture entry and exit. As a result, the annuloplasty deviates from the VBR and creates a waist sign (Fig. 3A). No coronary artery distortion or conduction abnormalities were observed in either group.

Due to the minimal elasticity of the CV-0 suture, theoretically speaking, the VBR is expected to have less expansibility during the cardiac cycle when it is closer to the annuloplasty plane, whereas greater deviations from the plane result in less influence from the suture and greater expansibility. It was demonstrated in our study that the internal group tended to have less VBR expansibility than the external group postoperatively (mean area variation, 4.9 ± 2.3% *vs.* 7.6 ± 4.1%, *p* = 0.078). Whether this difference can be translated into a lower rate of late annular redilatation needs to be confirmed by long-term follow-up studies. Moreover, our study revealed that the actual reduction in annular dimensions was more precise in the internal group than in the external group. The greater degree of discrepancy between the postoperative annulus dimensions and the size of the Hegar dilator in the external group were probably due to the external suture incorporating more septal muscle than the internal suture along the anterior annulus in the BAV, which led to asymmetrical circumferential annuloplasty and suboptimal VBR remodeling by the Hegar dilator [29]. A similar finding was reported by Holst *et al*. [23], who showed a larger VBR with external suture annuloplasty immediately after surgery and during follow-up than with internal suture annuloplasty, which was attributed to the larger baseline annulus in external annuloplasty patients rather than the type of annuloplasty used. Further research is needed to determine whether the rate of annular redilatation and long-term annular stability are affected by the different annuloplasty methods.

### Limitations

These findings need to be confirmed in a larger number of patients undergoing isolated BAV repair. Annular reduction strategies with the Hegar dilator differed between the two groups, which made a direct comparison of the postoperative annular reduction effect between the two annuloplasty techniques impossible. Our study had a single-center design and only immediate postoperative outcomes; to obtain more definite conclusions on long-term annulus stability after suture annuloplasty, a longer and more complete follow-up is required, incorporating the validation of MS-CT measurements by a core laboratory. A multicenter, prospective, randomized trial is required to minimize this bias.

## 5. Conclusions

In conclusion, internal suture annuloplasty resulted in better positioning relative to the VBR plane than external suture annuloplasty owing to the absence of VAJ interference. The short-term effect of more precise annular reduction with less expansibility obtained when using internal annuloplasty warrants long-term follow-up.

## Data Availability

All data generated or analyzed during this study are included in this article and its supplementary material files. Further enquiries can be directed to the corresponding author.

## Abbreviations

BAV, bicuspid aortic valve repair; AR, aortic regurgitation; VBR, virtual basal ring; VAJ, ventricular-annular junction; LVOT, left ventricular outflow tract; ECG, electrocardiogram; CT, computed tomography; RC, right coronary; LC, left coronary; NC, non-coronary; RN, right/non; LN, left/non; SD, standard deviation; MS, multislice.
